# Spatial patterns of fetal loss and infant death in an arsenic-affected area in Bangladesh

**DOI:** 10.1186/1476-072X-9-53

**Published:** 2010-10-26

**Authors:** Nazmul Sohel, Marie Vahter, Mohammad Ali, Mahfuzar Rahman, Anisur Rahman, Peter Kim Streatfield, Pavlos S Kanaroglou, Lars Åke Persson

**Affiliations:** 1 Department of Women's and Children's Health, International Maternal and Child Health (IMCH), Uppsala University, Uppsala, Sweden; 2 Institute of Environmental Medicine, Karolinska Institutet, Stockholm, Sweden; 3 International Vaccine Institute, Seoul, Korea; 4 ICDDR,B, Dhaka, Bangladesh; 5 School of Geography and Earth Science, McMaster University, Hamilton, ON, Canada

## Abstract

**Background:**

Arsenic exposure in pregnancy is associated with adverse pregnancy outcome and infant mortality. Knowledge of the spatial characteristics of the outcomes and their possible link to arsenic exposure are important for planning effective mitigation activities. The aim of this study was to identify spatial and spatiotemporal clustering of fetal loss and infant death, and spatial relationships between high and low clusters of fetal loss and infant death rates and high and low clusters of arsenic concentrations in tube-well water used for drinking.

**Methods:**

Pregnant women from Matlab, Bangladesh, who used tube-well water for drinking while pregnant between 1991 and 2000, were included in this study. In total 29,134 pregnancies were identified. A spatial scan test was used to identify unique non-random spatial and spatiotemporal clusters of fetal loss and infant death using a retrospective spatial and spatiotemporal permutation and Poisson probability models.

**Results:**

Two significant clusters of fetal loss and infant death were identified and these clusters remained stable after adjustment for covariates. One cluster of higher rates of fetal loss and infant death was in the vicinity of the Meghna River, and the other cluster of lower rates was in the center of Matlab. The average concentration of arsenic in the water differed between these clusters (319 μg/L for the high cluster and 174 μg/L for the low cluster). The spatial patterns of arsenic concentrations in tube-well water were found to be linked with the adverse pregnancy outcome clusters. In the spatiotemporal analysis, only one high fetal loss and infant death cluster was identified in the same high cluster area obtained from purely spatial analysis. However, the cluster was no longer significant after adjustment for the covariates.

**Conclusion:**

The finding of this study suggests that given the geographical variation in tube-well water contamination, higher fetal loss and infant deaths were observed in the areas of higher arsenic concentrations in groundwater. This illustrates a possible link between arsenic contamination in tube-well water and adverse pregnancy outcome. Thus, these areas should be considered a priority in arsenic mitigation programs.

## Background

Despite improvements in child survival, annually, more than nine million children die before the age of five, mostly in low- and middle-income countries [1,2]. About four million of these deaths are within the first four weeks of life and a similar number of stillbirths occur [1-3]. In Bangladesh, under-five mortality has rapidly declined [4], but the magnitude of the decrease varies geographically [5]. Improved primary health care including preventive services such as immunizations have contributed to this reduction, but still 65 out of 1000 live-born infants in Bangladesh die before one year of age [6].

Polluted drinking water is a common source of infant mortality [7]. After massive campaigns in the 1970s, people in Bangladesh started to use tube-well water instead of surface water for drinking as a measure for controlling cholera and other waterborne enteric diseases [7]. Tube-wells are long tubes (often open) drilled down about 20-100 m to extract groundwater and were primarily designed for irrigation purposes. Since the 1990s, excessive amounts of arsenic have been detected in the water from many of the tube-wells, which means many are contaminated with arsenic [8]. Arsenic is a toxic and carcinogenic substance that can be present in groundwater in differing levels of concentration [9,10] and tube-wells are used to extract ground water. Due to geological variation [11] and sedimentation processes in the Delta region in Bangladesh, arsenic laden soil carried from the Himalayas [12] have been deposited near the surface and then transported to deeper layers [13]. As a consequence, some areas have higher arsenic concentrations in their tube-well water than others.

Studies from Bangladesh and West Bengal [14-18] indicate arsenic exposure may increase the risk of low birth weight, fetal loss and, infant death. Even in the adult population in Bangladesh, chronic exposure to arsenic increases the risk of mortality due to infections and other causes [19]. Matlab, a rural area of Bangladesh, is one of the most severely affected arsenic areas, where 95% of the population use tube-well water for drinking [20]. In a study covering the period 1991-2000, Rahman and coworkers [14] observed 14% increased risk for fetal loss and 17% increased risk for infant death in pregnant women drinking tube-well water with arsenic concentrations above 50 μg/L (the Bangladesh drinking water standard). Clusters of high or low levels of human exposure to water with arsenic emanating from tube-well water have been identified [21]. Thus, it is relevant to explore relationship between spatial and spatiotemporal patterns of fetal loss and infant death with arsenic contaminated drinking water, as well as the spatial relationship between high and low clusters of fetal loss and infant death and high and low levels of arsenic concentration in tube-well water. Therefore, the study focused on detecting spatial and spatiotemporal clustering of fetal loss and infant mortality, and spatial relationships between high and low clusters of fetal loss and infant death rates and high and low levels of arsenic concentrations in tube-well water.

## Materials and methods

### Study area

The study was conducted in Matlab, a rural area of Bangladesh located 53 km southeast of Dhaka [14], which is a research site of the International Centre for Diarrhoeal Disease Research, Bangladesh (ICDDR,B). The ICDDR,B has maintained a Health and Demographic Surveillance System (HDSS) in Matlab since 1966 and covers a population of about 220,000. The HDSS surveillance area is divided into two parts, one with health services provided by the government and the other with health services provided by ICDDR,B.

### Study population

Community health research workers (CHRW) visit each household on a monthly basis to collect information on demographic events such as births, deaths, in-migration, out-migration, marriage, and pregnancy. Socioeconomic surveys (SES) are conducted every ten years and provide detailed socioeconomic information including individual educational level and household assets. The majority of the population lives in poor socioeconomic conditions and is mostly engaged in agricultural production. Three to six households, often related through the patrilineal line, form a *bari*, keeping a common courtyard in front of each household [21,22]. There is at least one tube-well for drinking purposes in each *bari*. In addition, they share other water sources, e.g. rivers, canals and ponds.

Pregnant women between 1991 and 2000 who drank water from the tube-wells tested in the 2002-2003 survey were included in the study [14]: this rendered 29,134 pregnant women available for analysis. The pregnant women were identified by CHRW during their monthly home visits and information on pregnancy outcome was collected on subsequent visits. Pregnancy outcomes were categorized as fetal loss (early and late), induced abortion and, live births. Early fetal loss was defined as loss of a fetus within 28 weeks of pregnancy and late fetal loss was defined as birth of a dead fetus after 28 weeks of gestation but did not include induced abortion. All live births were followed and any death among these births was recorded. Procedures for pregnancy detection and outcome measurements are described elsewhere [14]. An infant death was defined as a live birth dying before the age of one year. From the 29,134 pregnancies identified, 1,075 were excluded due to induced abortion, assuming no association of this outcome with arsenic exposure. In addition, 528 pregnancies were excluded due to missing household location in the GIS database and 559 pregnancies due to missing socioeconomic information. Finally, 26,972 pregnancies were included in the analysis. Arsenic concentration in the water was available for all functioning tube-wells (n = 6317) used by the pregnant women during their pregnancies.

Since 1994, the Geographic Information System (GIS) program of the ICDDR,B has been collecting, maintaining and regularly updating spatial information of Matlab to identify locations of *baris*, rivers, canals and, sources of drinking/cooking water (tube-wells, ponds, ditches, wells). Details are provided elsewhere [23]. The location of *baris *is regularly updated and integrated with the demographic surveillance. Locations of health facilities including hospital and sub centers for the community-based health programs are also available in the GIS database. A team of surveyors have measured the location of all functioning (13,286) and non-functioning tube-wells (3215) through the global positioning system (GPS) during an arsenic survey in 2002-2003. All functioning tube-wells have been tested for arsenic concentration in water: arsenic concentrations in these tube-wells range from < 1.0 μg/L to 3644 μg/L [24]. Each tube-well was assigned a *bari *identity code to link the tube-well characteristics to the population database. The study was approved by the ethical review committee of ICDDR,B, Dhaka, Bangladesh.

### Exposure assessment

Individual levels of water consumption history (including time periods) were obtained from the Arsenic and health consequences in Matlab (AsMat) survey conducted in 2002-2003 [24]. In the survey, a team of trained interviewers visited all houses with structured questionnaires and, asked about lifetime water consumption histories. Another team collected water samples from all active tube-wells, which were analyzed for arsenic concentration by Hydride Generation Atomic Absorption Spectrophotometry (HG-AAS; Shimudzu AA6800, Shimudzu Corporation, Kyoto, Japan) [25]. Arsenic exposure during pregnancy, based on a woman's reported source of drinking water at the time of pregnancy, was linked to outcome data.

### Possible confounders

Infant mortality in Matlab varies with socioeconomic conditions [26], education of the mother [27] and, parity. Arsenic exposure also differs among socioeconomic groups. Educated women or those with higher socioeconomic status have lower average arsenic exposure [24]. Information on mothers' education and household assets were obtained from the HDSS socioeconomic survey conducted in 1996 and data on pregnancies, pregnancy outcomes and, related covariates (age and parity) were obtained from the monthly updated HDSS databases. Each household's assets was assigned as a factor, scores were generated through principal component analysis and, categorized into quintiles [28] ranging from the lowest (poorest) to the highest (richest).

### Spatial analysis of arsenic concentrations and outcome

Several software packages incorporate techniques for detecting spatial patterns of health outcomes, disease cluster, and hotspots as well for developing spatial regression models [29]. In this study, local Moran's *I *statistic was used to detect high and low clusters of arsenic concentrations in tube-wells water and scan test to detect spatial and spatiotemporal clusters of fetal loss and infant mortality including the influence of confounding variables. The GIS database includes the location of *baris *linked to individual level information. Individual level pregnancy outcomes were aggregated by *bari*, the lowest identifiable geographic feature in the study area and, fetal loss and infant death were aggregated by *bari *for each year from 1991 to 2000. Similarly, average arsenic concentration in drinking water, women's educational level, and socioeconomic characteristics were aggregated by *bari *for each year of the study period. The people living in a *bari *were assumed to be homogeneous in their water usage and health behavior.

### Spatial clusters of arsenic concentrations

Arsenic concentration in water was available for all functioning tube-wells used and the locations of the tube-wells were linked to the population database for mapping spatial patterns of arsenic exposure among Matlab residents. For this analysis, a set of Thiessen polygons were created around the centroid of tube-wells, i.e. space is allocated for each tube-well (a GIS technique). Then local Moran's *I *statistic was calculated in Geoda (freeware, version 0.9.5i, Geoda Center, Arizona State University, USA) to detect high-high (high surrounded by high) and low-low (low surrounded by low) clusters of tube-well water arsenic concentration [30]. This required a spatial proximity matrix that provides information on the configuration and relative location of the polygons. Geoda allows for derivation of such a matrix.

### Spatial clusters of fetal loss and infant death

The spatial scan test has been widely used to detect potential geographic clusters of human diseases [31-34] and, is even suitable for uneven geographic distribution of cases and population density [35]. Spatial scan test was implemented in SaTScan® [31] to identify unique non-random space-time clusters through a retrospective space-time permutation model. SaTScan® can detect probable space and space-time locations including multiple clusters in a defined geographic area [32,33,36] through the uses of circles or ellipses as search windows and includes a non-parametric test statistic.

Fetal loss and infant mortality were assumed to have a Poisson distribution. Under the null hypothesis, fetal loss and infant death in a particular location is proportional to the number of pregnancy outcomes in that location [35]. With SaTScan®, the probability of the frequency of fetal loss and infant death at each peak surpassing that expected by chance was estimated. Space and time limitations were set to 50%, which allowed a scan for both large and small clusters. Thus, the maximum cluster size can be 50% of the total population and 50% of the total time, i.e. five years in this study, and takes into account the observed number of fetal losses and infant deaths inside and outside the search window when calculating the highest likelihood for each window [37]. This window was the most probable cluster and, had a rate that was the least likely to happen by chance alone. Purely spatial and spatiotemporal analyses were performed with circular search windows and, both unadjusted and with adjustment for covariates (education, SES, age and parity). The statistical significance (*p <*0.05) of possible clusters was calculated with 999 Monte Carlo simulations [38]. The output from SaTScan® was imported into the SPSS software (Version 16.0, Chicago, Illinois, USA) in order to evaluate differences in arsenic concentration between higher and lower risk clusters. A portion of the output was imported into ArcGIS (Version 9.2, ESRI, California, USA) to map significant (*p *≤ 0.10) clusters of higher and lower risk of fetal loss and infant mortality.

## Results

### Background data

The average age at pregnancy was 27 years (median: 26, inter-quartile range (IQR): 23-31 years) and half of the mothers (50%) had no formal education (zero years of schooling). The average arsenic concentration in drinking water during pregnancy was 241 μg/L (median: 226, IQR: 38-373). The rates of fetal loss and infant mortality increased with increasing arsenic exposure, decreased over time, and were higher for women below 20 years age or above 35 years of age (Table 1). Fetal loss and infant death were higher for women in their first pregnancy (data not shown). Women with low socioeconomic status or low educational attainment had higher fetal loss and infant death (χ^2 ^= 24.4, ρ < 0.001) and higher exposure to arsenic (F = 4.1, ρ = 0.003, Table 1). Therefore, the year of pregnancy outcome, age, parity, education and socioeconomic status were considered as potential confounders in the association between arsenic exposure and fetal loss or infant death.

**Table 1:** Distributions of maternal age, education, socioeconomic status and arsenic exposure by outcome (fetal loss and infant death vs. surviving and migration-out), Matlab, Bangladesh, 1991-2000

Variables	Fetal loss and infant death		Live infants and migration-out		Total	
	
	N	%	N	%	N	%
Age categories, years^a ^(χ^2 ^= 143.2, ρ < 0.001)						
< 20 20-24 25-29 30-34 > = 35	476 1037 1044 582 526	17 13 12 12 20	2276 7151 7594 4127 2159	83 87 88 88 80	2752 8188 8638 4709 2685	10 30 32 18 10
Education^b ^(χ^2 ^= 12.0, ρ = 0.007)						
No education Primary Secondary Higher	1933 1032 655 45	14 13 12 12	11519 6825 4626 337	86 87 88 88	13452 7857 5281 382	50 29 20 1
Household assets score quintiles (χ^2 ^= 15.5, ρ = 0.004)						
1 (poor) 2 3 4 5 (rich)	668 839 747 741 670	14 15 14 13 13	3975 4875 4775 5107 4575	86 85 86 87 87	4643 5714 5522 5848 5245	17 21 21 22 19
Arsenic concentration in tube-well water^c ^(μg/L) (χ^2 ^= 24.4, ρ < 0.001)						
< 10 10-49 50-199 200-299 300-399 400-499 > = 500	652 209 672 720 588 406 418	12 12 14 14 15 15 14	4684 1572 4302 4386 3361 2327 2675	88 88 86 86 85 85 86	5336 1781 4974 5106 3949 2733 3093	20 7 18 19 15 10 11
Year of pregnancy outcomes (χ^2 ^= 21.9, ρ < 0.001)						
1991-1992 1993-1994 1995-1996 1997-1998 1999-2000	637 679 682 784 883	15 15 13 13 13	3495 4002 4530 5274 6006	85 85 83 83 83	4132 4681 5212 6058 6889	15 17 19 23 26

^a ^Age at pregnancy in years, mean: 27 (SD: 5.8), median: 26 and inter-quartile range: 23-31

^b ^Mothers education in years, mean: 2.9 (SD: 3.4), median: 1.0 and inter-quartile range: 00-5.0

^c ^Arsenic exposure during pregnancy in μg/L, mean: 241 (SD: 209), median: 226 and inter-quartile range: 38-373

### Spatial analysis of arsenic concentration

**Figure 1:**
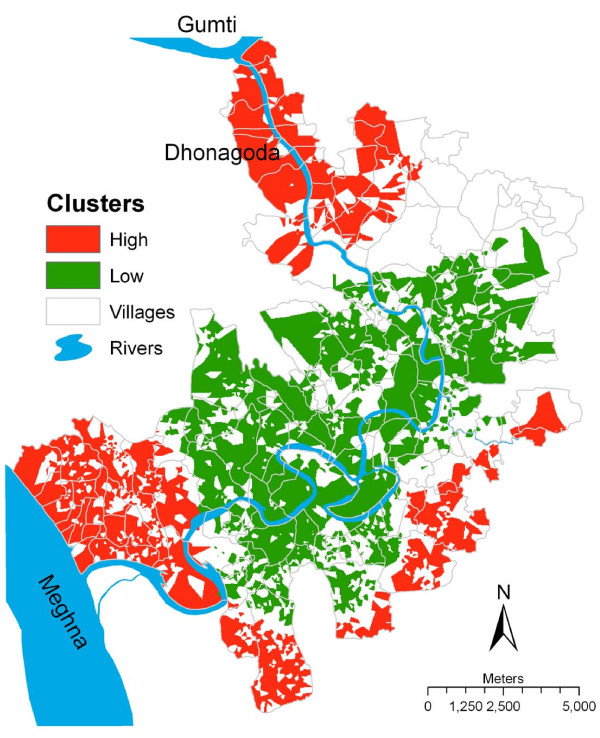
**The high and low clusters of arsenic exposure in Matlab, Bangladesh**. Clusters were detected through arsenic concentration in tube-well water used by the pregnant women during pregnancy in 1991-2000.

Arsenic concentrations in water of the functioning tube-wells (n = 6317) used by the pregnant women and the location of these tube-wells were used to identify clusters of arsenic exposure. Only the significant clusters of tube-well water arsenic with high concentration areas surrounded by high concentration areas (high-high) or low concentration areas surrounded by low concentration areas (low-low) were considered. High arsenic concentration clusters were identified in the north and in the southwest of Matlab, and large low arsenic concentration clusters were detected at the center of the study area (Figure 1). High arsenic concentration clusters were also detected in the south and from south to southeast of Matlab. All clusters were small in size i.e. area delimited by the Thiessen polygon around individual tube-wells and comprised several small clusters.

### Spatial analysis of fetal loss and infant death rates

**Figure 2:**
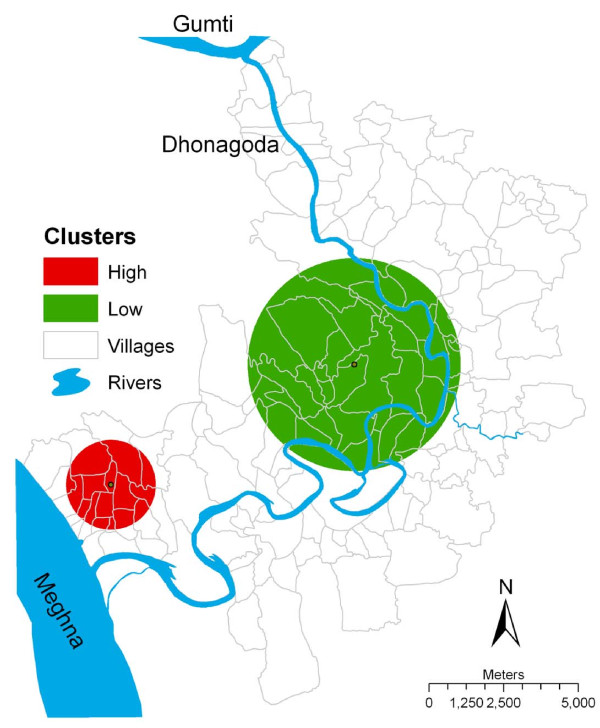
**The clusters of significantly higher or lower fetal loss and infant death in Matlab, Bangladesh, 1991-2000**. Cluster size, shape and locations remained the same after adjustment for covariate (age, parity, SES and, education), and is why unadjusted clusters are presented.

In the spatial analysis, two clusters with significantly different rates of fetal loss and infant death were identified, as compared to the expected numbers: one with lower and the other with higher occurrence than expected under the null hypothesis. The cluster of higher occurrence was found in southwest of Matlab (Figure 2) and had 314 observed cases (expected was 229) and a relative risk of 1.41, indicating a 41% higher risk of fetal loss and infant death than for other pregnancies outside the cluster (Table 2). The statistically significant low risk cluster was detected in the central part of Matlab, where 429 cases (expected 545) of fetal loss and infant death in the cluster yielded a relative risk of 0.76, indicating 24% lower risk of fetal loss and infant death inside the cluster than outside the cluster.

**Table 2:** Significant spatial and spatiotemporal clusters of fetal loss and infant death in Matlab, Bangladesh, 1991-2000

Analysis type	Adjustment	Cluster	Time	Observed cases	Expected cases	Relative Risk	P value	Arsenic concentration		
								
								Mean	Median	IQR
*Spatial*	Unadjusted	High	-	314	228.55	1.409	0.005	319	312	171-433
		Low	-	429	545.33	0.758	0.004	174	74	1-302
										
	Adjusted*	High	-	303	218.98	1.418	0.005	322	312	181-436
		Low	-	429	541.75	0.764	0.008	174	74	1-302
										
*Spatiotemporal*	Unadjusted	High	1992-1995	294	209.73	1.437	0.019	322	304	186-435
		Low	-	-	-	-	-	-	-	-
				-	-	-	-	-	-	-
	Adjusted*	High	-	-	-	-	-	-	-	-
		Low	-	-	-	-	-	-	-	-

IQR: Inter Quartile Range,

* Analysis was adjusted for age, parity, education and socioeconomic status

**Figure 3:**
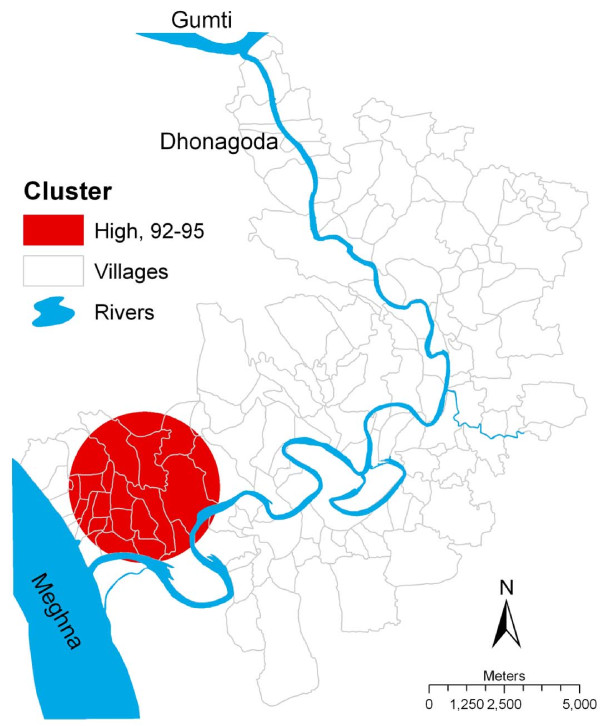
**Spatiotemporal cluster of fetal loss and infant death, Matlab, Bangladesh, 1991-2000. The clusters were obtained from the unadjusted analysis**.

Note, Unadjusted analysis and adjustment for age, parity, education and socioeconomic status are presented separately. Arsenic concentration via tube-well water was compared between clusters by Analysis of variance (ANOVA). (See Figures 2 and 3 for cluster location).

The average arsenic concentration in the tube-well water used by the women in the clusters of higher risk for fetal loss and infant death was 319 μg/L, in the cluster of lower risk this was 174 μg/L: the difference was statistically significant (p < 0.001). The results of the spatial analysis of fetal loss and infant death remained the same after inclusion of the covariates (age, parity, education and, socioeconomic status) in the model (Table 2, Figure 2) and, the difference in average arsenic concentration during pregnancy between high and low risk clusters remained statistically significant (p < 0.001).

When the clusters of fetal loss and infant death were superimposed onto the clusters of arsenic contamination in tube-well water, the high risk cluster of fetal loss and infant death corresponded with the cluster of high arsenic concentrations in the tube-well water in the southwest, and the low risk cluster of fetal loss and infant death corresponded with the cluster of low arsenic concentrations in the tube-well water (Figures 1 and 2). The high risk cluster of arsenic contaminations detected in the tube-well water in the north of Matlab did not correspond to the high risk cluster of fetal loss and infant death.

### Spatiotemporal analysis of fetal loss and infant death rates

The results of the spatiotemporal analysis of fetal loss and infant death rates from 1991 to 2000 are presented in Table 2 and Figure 3. A significant high risk cluster of fetal loss and infant death rates was observed for the period 1992 to 1995 with a relative risk of 1.44, indicating a 44% increased risk of fetal loss and infant death inside the cluster than outside the cluster. However, no significant low risk cluster of fetal loss and infant death rates was identified in the spatiotemporal analysis. Compared to the high risk cluster of fetal loss and infant death rates obtained from purely spatial analysis, the spatiotemporal cluster of fetal loss and infant death rates was more to the northeast of Matlab and expanded the size of cluster. In the spatiotemporal analysis, the average arsenic concentration in tube-well water during the significant period of study (1992-1995) was 322 μg/L for the high risk cluster, which was similar to that in the purely spatial analysis. When age, parity, education and socioeconomic status were included in the model, the high-risk spatiotemporal cluster for arsenic concentration in tube-well water was no longer statistically significant.

## Discussion and conclusion

This study identified one cluster of significantly higher rates of fetal loss and infant death in Matlab after being adjusted for potential confounders. This may be due to higher arsenic concentration in the tube-well water, and specifically corresponded to hot spots of elevated arsenic concentrations in groundwater in those tube-wells. One cluster of significantly lower rates of fetal loss and infant death was identified, which was positively related to the cluster of lower arsenic concentrations in tube-wells. As there are earlier reports on epidemiological relationship [14-18] between prenatal arsenic exposure and fetal loss and infant mortality, the results of this analysis suggested extensive arsenic concentration in groundwater contributed to the clustering of adverse pregnancy outcome and infant mortality in this area. This data could contribute to the development effective plans for continued mitigation activities.

One of the strengths of this study was that it was population-based and conducted in an area where ICDDR,B has maintained a surveillance system for more than four decades [39]. The health and demographic information is linked to a geographic information system and continuous update of the surveillance system is managed by CHRW who collect demographic information on a monthly basis that is entered into the population database. To ensure data quality, 5% of the demographic events are rechecked by their supervisors [39]. Information on pregnancy and pregnancy outcomes were prospectively collected by CHRW during their monthly home visit. Although there is a risk of underestimating the occurrence of early pregnancy losses in this data collection system, late pregnancy losses and the occurrences of infant death were accurately reported.

It is unlikely the pregnancies that were excluded due to missing or destroyed tube-wells affected the results of this analysis, as these cases were randomly distributed in space. One potential limitation was the measurement of arsenic contamination in tube-well water, which was done in 2002-2003, while the period of analysis for fetal loss and infant death was from 1991-2000. Arsenic concentration in tube well water is stable over time [40,41], and 60 tube wells in Matlab area have been followed-up three times per year over three years with no major changes in arsenic concentrations [14], which supports other findings. Thus, the information on arsenic concentration in tube-well water derived from 2002-2003 data combined with water exposure history was considered a valid representation of arsenic concentrations in tube-well water for this adverse pregnancy outcomes analysis.

The clusters were checked by including household level socioeconomic status and mother education as the covariates in the spatial model. The result of the spatial model did not alter the outcomes. This results of this analysis are supported by the findings of Rahman et al [14], who, in their individual level analysis observed insignificant effects of education and socioeconomic status on the risk for fetal loss and infant deaths in the presence of arsenic exposure. However, the study was designed to analyze the spatial cluster of arsenic concentration and to evaluate clusters of fetal loss and infant death rather than to undertake an assessment of individual risks related to prenatal arsenic exposure.

Two of the major rivers in Bangladesh, the Ganges and the Brahmaputra, originate from the Himalayas and merge in the northwest of Matlab to become the largest river of Bangladesh, the Meghna River. Another major river, the Gumti, running from the northeast, touches the north tip of Matlab sub-district and merges into the Meghna. A small river, Dhonagoda, runs through Matlab, connecting the Gumti and Meghna rivers. The complex structure of this delta land renders it difficult to fully determine the sedimentation processes and associated arsenic contamination of soil and ground water and the spatial variation in a small area. Two big clusters of high arsenic concentrations in tube-wells water were observed in the north and southwest of Matlab along with several small clusters in the south and southeast of the study area along the study area boundary. However, there could be uncertainty about the clusters near the study area boundary due to low number of observations in that area [29,42]. The clusters of higher arsenic concentrations in tube-well water in the north and in the southwest could be due to the effect of the sedimentation process in those areas. However, arsenic contamination decreased with increasing distance from the major rivers so that a cluster of lower arsenic exposure formed in the central part of Matlab.

Individual level arsenic exposure is related to increased risk of fetal loss, infant death, low birth weight and several other adverse health consequences [14-18]. Therefore, it was logical to expect an association between the areas with elevated arsenic contamination in groundwater (that have a variation in space) and spatial clustering of fetal loss and infant mortality. Only one of the detected clusters of higher arsenic contamination in tube-well water, in the southwest of Matlab, corresponded with a significantly high cluster of fetal loss and infant death near the confluence of the Meghna and Dhonagoda Rivers. However, the fetal loss and infant deaths were higher in the high-arsenic contamination in tube-well water area in the north of Matlab, although this cluster was not statistically significant.

A number of mitigation activities were initiated in Matlab in 2002-2003 in collaboration with a non-governmental organization, Bangladesh Rural Advancement Committee (BRAC). The mitigation program informed about arsenic concentrations in the tube-well water and, marked tube-wells with arsenic concentrations exceeding the national standard (50 μg/L) with a red color. Additionally, the program assisted people in obtaining arsenic-free drinking water sources, e.g. treated surface water (pond sand filters), harvesting of rain water and alternative ground water sources after screening and identifying arsenic contaminated tube-wells [8,43]. However, a recent study from Matlab [44] revealed children are still exposed to arsenic contamination in tube-well water for drinking, indicating the mitigation programs fail to provide adequate safe water to the people of the area. The finding of this study suggests that given the geographical variation in arsenic concentration in tube-well water, higher fetal loss and infant deaths were observed in the areas of higher arsenic concentrations that illustrates a possible link between arsenic concentration in tube-well water and adverse pregnancy outcome. Thus, these areas with hotspots of arsenic concentration in tube-well water should be considered a priority in arsenic mitigation programs for providing safe, arsenic free drinking water.

## Competing interests

The authors declare that they have no competing interests.

## Authors' contributions

NS participated in the study design, analyzed data, interpreted the results, and drafted the manuscript. LAP, MV and MA participated in the study design, made significant revisions and contributed to the manuscript. MR, AR, PKS and PSK made significant contributions to the manuscript. All authors read and approved the final manuscript.
